# Identification and Validation of a PPP1R12A-Related Five-Gene Signature Associated With Metabolism to Predict the Prognosis of Patients With Prostate Cancer

**DOI:** 10.3389/fgene.2021.703210

**Published:** 2021-08-13

**Authors:** Zhihao Zou, Ren Liu, Yingke Liang, Rui Zhou, Qishan Dai, Zhaodong Han, Minyao Jiang, Yangjia Zhuo, Yixun Zhang, Yuanfa Feng, Xuejin Zhu, Shanghua Cai, Jundong Lin, Zhenfeng Tang, Weide Zhong, Yuxiang Liang

**Affiliations:** ^1^Department of Geriatrics, The Second Affiliated Hospital of South China University of Technology, Guangzhou, China; ^2^Guangdong Key Laboratory of Clinical Molecular Medicine and Diagnostics, Department of Urology, School of Medicine, Guangzhou First People’s Hospital, South China University of Technology, Guangzhou, China; ^3^Guangdong Provincial Institute of Nephrology, Nanfang Hospital, Southern Medical University, Guangzhou, China; ^4^Department of Urology, Huadu District People’s Hospital, Southern Medical University, Guangzhou, China; ^5^Urology Key Laboratory of Guangdong Province, The First Affiliated Hospital of Guangzhou Medical University, Guangzhou Medical University, Guangzhou, China; ^6^State Key Laboratory of Quality Research in Chinese Medicines, Macau University of Science and Technology, Macau, China; ^7^Department of Urology, Huizhou Municipal Central Hospital, Huizhou, China

**Keywords:** prostate cancer, protein phosphatase 1 regulatory subunit 12A, metabolism, gene signature, prognostic model

## Abstract

**Background:**

Prostate cancer (PCa) is the most common malignant male neoplasm in the American male population. Our prior studies have demonstrated that protein phosphatase 1 regulatory subunit 12A (PPP1R12A) could be an efficient prognostic factor in patients with PCa, promoting further investigation. The present study attempted to construct a gene signature based on PPP1R12A and metabolism-related genes to predict the prognosis of PCa patients.

**Methods:**

The mRNA expression profiles of 499 tumor and 52 normal tissues were extracted from The Cancer Genome Atlas (TCGA) database. We selected differentially expressed PPP1R12A-related genes among these mRNAs. Tandem affinity purification-mass spectrometry was used to identify the proteins that directly interact with PPP1R12A. Gene set enrichment analysis (GSEA) was used to extract metabolism-related genes. Univariate Cox regression analysis and a random survival forest algorithm were used to confirm optimal genes to build a prognostic risk model.

**Results:**

We identified a five-gene signature (*PPP1R12A*, *PTGS2*, *GGCT*, *AOX1*, and *NT5E*) that was associated with PPP1R12A and metabolism in PCa, which effectively predicted disease-free survival (DFS) and biochemical relapse-free survival (BRFS). Moreover, the signature was validated by two internal datasets from TCGA and one external dataset from the Gene Expression Omnibus (GEO).

**Conclusion:**

The five-gene signature is an effective potential factor to predict the prognosis of PCa, classifying PCa patients into high- and low-risk groups, which might provide potential novel treatment strategies for these patients.

## Introduction

Prostate cancer (PCa) is the most common male malignancy in the developed world and is predicted to account for ∼26% of new cancer diagnoses among US men and 14.1% worldwide in 2021 (Ferlay et al., 2020; Siegel et al., 2021). In cases with early detection, radical prostatectomy (RP), in which local malignant prostate tissue is resected, is the preferred method to treat PCa patients. However, the high recurrence rates of PCa contribute to the risks of progression to castration-resistant PCa (CRPC), giving rise to the second-leading cause of cancer deaths (Ward et al., 2003; Roehl et al., 2004; Ferlay et al., 2018; Matsumoto et al., 2018). Potent diagnostic and prognostic biomarkers are necessary to predict the course of disease and the medical therapeutic efficacy in personalized medicine (Huber et al., 2015). It is demonstrated that the most commonly used clinicopathological factors used to provide important prognostic information for monitoring disease progression in PCa are serum prostate-specific antigen (PSA) level, the Gleason score, the pathological tumor stage, and the surgical margin (Quinn et al., 2001). Nevertheless, because of the great heterogeneity of PCa, the prediction power of these conventional markers is often not satisfactory, and the prognostic markers have not been fully explored yet. Therefore, identification of novel biomarkers for PCa to enhance the accuracy of PCa aggressiveness prediction still is a significant task.

Protein phosphatase 1 regulatory subunit 12a (PPP1R12A), also named myosin phosphatase targeting subunit 1 (MYPT1), is a member of the myosin phosphatase-targeting (MYPT) protein family and participates in the regulation of smooth muscle contraction (Murthy, 2006; Rattan et al., 2010; He et al., 2013; Qiao et al., 2014). In addition, the functions of PPP1R12A in cell development (Weiser et al., 2009), the cell cycle (Yamashiro et al., 2008; Dumitru et al., 2017), and cell adhesion and migration (Joo and Yamada, 2014) have been observed recently in accumulating studies. Importantly, PPP1R12A could inhibit angiogenesis and tumor growth and predict aggressive outcomes in PCa (Lin et al., 2017). Moreover, its expression in PCa, combined with CD31, could be a significant prognostic factor (Liang et al., 2018). Therefore, it is essential to explore more biomarkers that interact with PPP1R12A for the early effective prognosis, and effective treatment of PCa.

After the discovery of the Warburg Effect (Warburg, 1956), metabolic reprogramming drew increased attention in the cancer field and has since become a novel hallmark of cancer (Hanahan and Weinberg, 2011). Anabolic and catabolic metabolism are indispensable to cancer cells in metabolic reprogramming, ensuring that the biomass synthesis and energy supply of cancer cells are adequately supplied (Ward and Thompson, 2012; Pavlova and Thompson, 2016; Sun et al., 2018; Cardoso et al., 2021). Previous studies have revealed a tremendous difference in metabolic statues between tumors and normal tissue due to the unlimited proliferative nature of cancer cells (El Hassouni et al., 2020). However, little is known about the metabolic microenvironment and its prognostic value in PCa. It is particularly significant to recognize biomarkers with high specificities and sensitivities in consideration of the prognosis of PCa at the metabolic level.

In the present study, we extracted five genes related to PPP1R12A interaction and metabolism and developed a robust five-gene signature to predict the prognosis of PCa. Our findings provide a new perspective for the development of therapeutical strategies and personalized treatment approaches.

## Materials and Methods

### Collection of Human Tissue Samples

The patient cohorts and tissue samples in this study were the same as those used in our previous study (Lin et al., 2017; Liang et al., 2018). In total, 225 consecutive PCa patients that underwent radical prostatectomy were included in the human PCa tissue microarrays (TMA). Related clinicopathological data were included.

### UALCAN Analysis

The UALCAN database, a publicly accessible web portal that is easy to operate,^1^ includes clinical data from 31 cancer types and is commonly used to investigate relative transcriptional expression levels between tumor and normal samples. *PPP1R12A* expression was identified using the “prostate adenocarcinoma” dataset with the “Expression Analysis” module. Expression levels of *PPP1R12A* across PCa and normal samples as well as the relationships between *PPP1R12A* and different Gleason scores were analyzed. *p* < 0.05 was regarded significant.

### Direct PPP1R12A Protein Interactor Analysis

The PCa cell line LNCaP, obtained from the American Type Culture Collection (United States), was maintained in RPMI 1640 medium (Hyclone, United States) supplemented with 10% fetal bovine serum (Gibico, United States) and 1% penicillin–streptomycin at 37°C in 5% CO2. LNCaP cells were transfected with a PPP1R12A-encoding vector construct and screened with puromycin. After clone selection, using full-length PPP1R12A protein as bait, we performed tandem affinity purification–mass spectrometry (TAP-MS) (Li et al., 2016) to identify direct PPP1R12A interactors on a proteomic scale. We utilized the Protein Pilot 5.0 software and MASCOT software to identify putative PPP1R12A-binding motifs in interaction partners (Perkins et al., 1999).

### Data Processing

The Cancer Genome Atlas (TCGA)^2^ contains both sequencing and pathological data for 30 different cancers (Cancer Genome Atlas Network, 2012). We acquired the FPKM data of TCGA RNA-seq datasets for PCa from the UCSC Xena browser.^3^ We changed the type of gene expression profiles from log_2_(FPKM + 1) to log_2_(TPM + 1) to obtain a more precise data of differentially expressed genes (DEGs). The clinical information for PCa was acquired from cBioPortal.^4^ Moreover, gene expression profiles from the Taylor (GSE21034) (Taylor et al., 2010) and GSE6956 (Wallace et al., 2008) datasets were retrieved from GEO.^5^ The expression profiles data from the Taylor dataset were standardized through RMA and data from the GSE6956 dataset were standardized through MAS5.

### Differentially Expressed Gene and Metabolism-Related Genes Analysis

DEGs were identified among the 499 tumor samples and 52 normal prostate gland tissues after normalization. Based on the expression levels of PPP1R12A, the patient samples were ranked from high to low. Thereafter, we used the median expression of PPP1R12A (4.3584) as cut-off value, and the samples data were classified into two groups, the high expression group and the low expression group. We utilized the “Limma” package (Ritchie et al., 2015) in R (version 3.6.0) to screen the DEGs related to PPP1R12A using the screening criteria of adjusted *p* < 0.01 (Benjamini and Hochberg; Madar and Batista, 2016) and | log_2_(fold change)| ≥ 1. The “pheatmap” package was applied for clustering analyses and heatmaps plotting.

We acquired metabolic pathway gene sets from the Molecular Signatures Database^6^ (MSigDB) (Subramanian et al., 2005) and the Kyoto Encyclopedia of Genes and Genomes (KEGG) (Ogata et al., 1999). Using the software of GSEA v4.0.3 for Windows and the “c2.cp.kegg.v7.0.symbols.gmt” gene set, the metabolism-related genes were defined as the genes that were enriched in metabolic pathways by calculating the PCa-related activity levels.

### Functional Enrichment Analysis

The elemental biological functions of PPP1R12A-related DEGs in PCa can be assessed by performing Gene Ontology (GO) (The Gene Ontology Consortium, 2019) enrichment analysis in three categories (biological processes, cellular component, and molecular function) and KEGG pathway enrichment analysis, which were conducted using the by R package (version 3.6.0) “clusterProfiler” (Yu et al., 2012; Kanehisa and Sato, 2020) with conditions of adjusted *p* < 0.05.

### Prognostic Gene Signature Screening and Generation

We utilized the open source toolkit scikit-learn^7^ in Python libraries (version 3.6.0) to select the most promising genes to establish and optimize the predictive random forest model. While constructing the random forest, we conducted sequencing for a total of 10 times, identifying the top five genes of the sequencing results. Subsequently, the genes whose occurrence frequency was greater than or equal to 5 were selected as genes of interest (Hastie et al., 2004).

### Construction and Validation of Prognostic Signature

The impact of each promising gene on disease-free survival (DFS) was explored by utilizing the univariate Cox regression analysis. According to the expression levels of screened genes, the risk score (RS) model was developed through Cox univariate analysis as the following formula: RS = (–0.4619 × expression value of *PTGS2*) + (–0.141 × expression value of *PPP1R12A*) + (–0.2915 × expression value of *NT5E*) + (–0.6051 × expression value of *AOX1*) + (0.3527 × expression value of *GGCT*). On the basis of the matching median RS, PCa patients were split into high- or low-risk groups. Using the RS-model formula, we calculated the RS for each patient in the TCGA internal validation cohort and one more external validation cohort to confirm the robustness of the prognostic gene signature. The elementary endpoint was DFS and the secondary endpoint was biochemical relapse-free survival (BRFS). Kaplan–Meier curve analysis was performed in the training set and validation set to assess the relationship between the RS and DFS as well as BRFS. We used receiver operating characteristic (ROC) curves to analyze the specificity and sensitivity of the constructed survival prediction classifier. The area under the ROC curve (AUC) value was calculated and compared to assess the classifier performance. *p* < 0.05 was considered to be statistically significant.

### Validation of Expression Levels and Subgroup Analysis of Screened Genes

To validate the expression characteristics of the five promising genes between the PCa and control groups, we analyzed the expression levels of the five genes obtained from the TCGA, Taylor, and GSE6956 datasets and depicted these using boxplots. Subgroup analysis in the TCGA cohort was conducted to validate the predictive superiority of the five-gene signature.

### Immunohistochemistry Analysis

Protein expression levels of PPP1R12A protein in PCa and benign tissues were tested by immunohistochemistry (IHC) as previously described.

### Statistical Analysis

All statistical analyses used were conducted using R software (version 3.6.0). Differential mRNA expression of PPP1R12A in PCa tissues from the TCGA database was tested using Student’s *t*-test. Functional enrichment of PPP1R12A-related DEGs in GO terms or KEGG pathways was analyzed using a hypergeometric test. To establish the risk assessment formula, Cox’s regression coefficient was obtained through univariate regression analyses. Survival curves were generated and compared by the log-rank test. *p* < 0.05 was considered statistically significant.

## Results

### PPP1R12A Expression Was Downregulated in PCa

**FIGURE 1:**
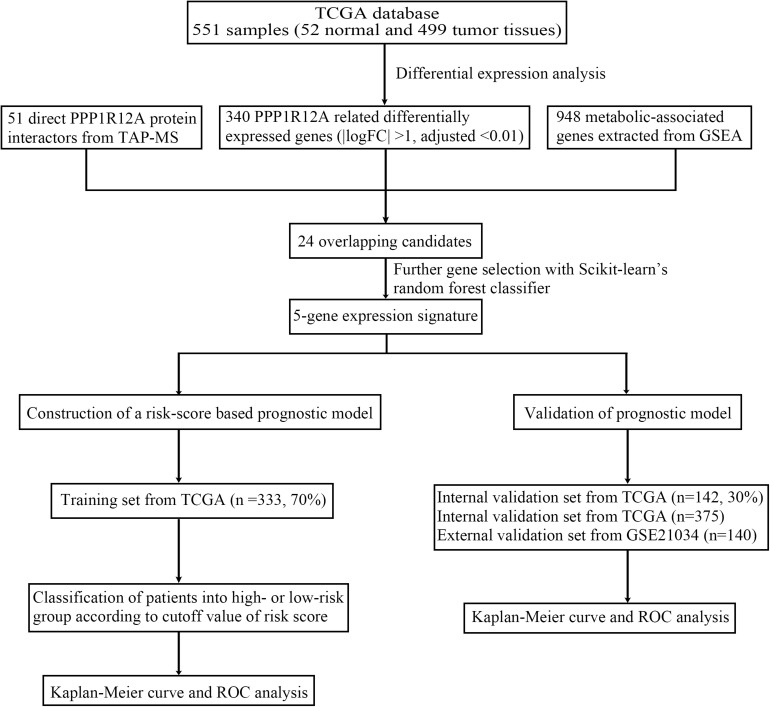
Flow chart of the study.

**FIGURE 2:**
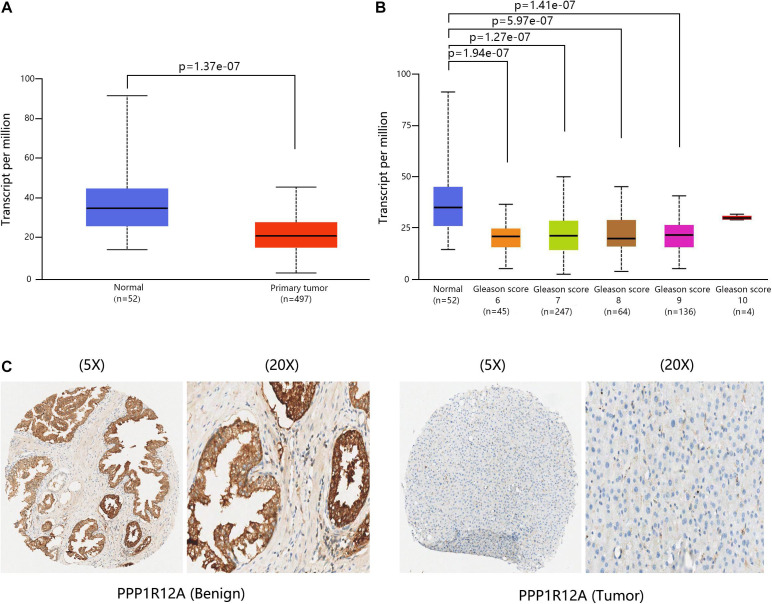
PPP1R12A was downregulated in PCa. **(A)** Relative levels of PPP1R12A in normal prostate and PCa samples based on the UACLAN database. **(B)** Boxplot showing the relative expression of PPP1R12A in healthy controls and PCa patients with different Gleason scores based on the UACLAN database. **(C)** IHC results for MYPT1 in benign and PCa tissues.

The overall design of our study is shown in Figure 1. In our previous study (Lin et al., 2017; Liang et al., 2018), we demonstrated that the combination of miR-30d/PPP1R12A and the combination of PPP1R12A/CD31 could be effective prognostic factors for human PCa. In order to analyze the expression profiles of PPP1R12A in PCa, mRNA expressions levels of *PPP1R12A* were analyzed by UALCAN, as revealed in 497 PCa samples in TCGA. As shown in Figure 2A, mRNA expression levels of *PPP1R12A* were significantly downregulated in PCa tissues compared to normal samples (*p* < 0.001). Furthermore, mRNA levels of *PPP1R12A* in PCa were significantly lower compared to the normal tissues in the subgroup analysis based on the Gleason score (Figure 2B). To further validate the level of PPP1R12A in PCa, we conducted IHC to explore the protein expression level of PPP1R12A in PCa and benign tissues. As shown in Figure 2C, the immunostaining of PPP1R12A protein in benign prostate tissues was markedly stronger than that in PCa tissues, indicating that the protein expression of PPP1R12A was lower in PCa than in normal prostate tissues. These results suggested that PPP1R12A was significantly downregulated in PCa relative to associated normal tissues.

### Identification of Direct PPP1R12A Protein Interactors and PPP1R12A-Related Differentially Expressed Genes

To obtain the direct human PPP1R12A protein interactome, we performed TAP-MS analyses with full-length PPP1R12A protein as bait. We further analyzed our dataset using Protein Pilot 5.0 software and MASCOT software to analyze PPP1R12A interactors, generating a final list of 51 PPP1R12A interactive proteins (Supplementary Table 1).

**FIGURE 3:**
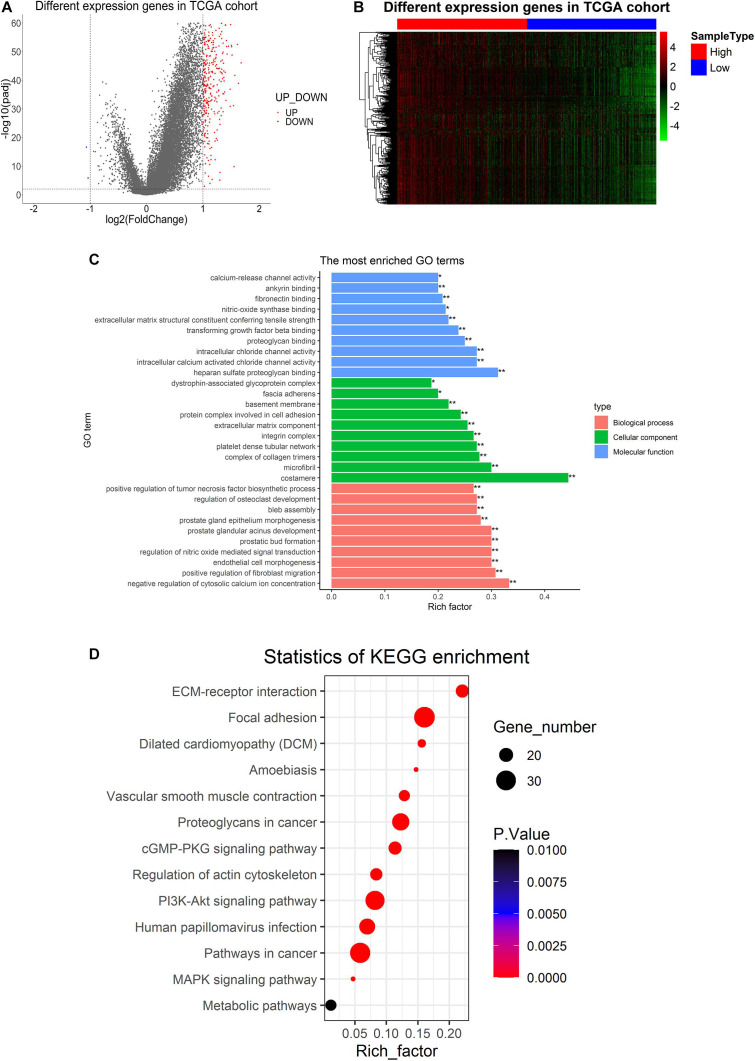
Differentially expressed genes in PCa tissues and GO and KEGG pathway analysis of PPP1R12A-related DEGs. **(A)** Volcano plot for the 340 DEGs from the TCGA PRAD dataset. Red indicates upregulation while blue indicates downregulation. **(B)** Heatmap plot of the top 340 DEGs from the TCGA PRAD dataset. The red shade represents high PPP1R12A expression tissue; the blue shade represents low PPP1R12A expression tissue. **(C,D)** GO functional enrichment analysis **(C)** and KEGG pathway analysis **(D)** of PPP1R12A-related DEGs. *P < 0.05, **P < 0.001.

Analysis of PPP1R12A-related DEGs was conducted between the high expression group (higher than median) and low expression group (lower than median) based on the predefined cut-off values (4.3584). With the screening criteria of *p* < 0.01 and | log (fold change) | > 1, we identified 340 PPP1R12A-related DEGs in the TCGA database after screening, which included 338 upregulated genes and two downregulated genes (Figure 3A). The heatmaps of these top differentially upregulated and downregulated PPP1R12A-related DEGs are shown in Figure 3B.

### Gene Functional Enrichment Analysis

GO molecular function enrichment analysis and KEGG pathway enrichment analysis were performed to analyze functional enrichment in PPP1R12A-related DEGs. The top 10 (^∗^*p* < 0.05; ^∗∗^*p* < 0.001) most significant GO terms are shown in Figure 3C, which reveals that the most common biological processes among the DEGs were negative regulation of cytosolic calcium ion concentration, positive regulation of fibroblast migration, tumor necrosis factor biosynthesis, endothelial cell morphogenesis, regulation of nitric oxide-mediated signal transduction, prostatic bud formation, prostate glandular acinus development, prostate gland epithelium morphogenesis, bleb assembly, and osteoclast development. Costamere, microfibril, complex of collagen trimers, platelet dense tubular network, integrin complexes, extracellular matrix (ECM) components, protein complexes involved in cell adhesion, basement membrane, fascia adherens, and dystrophin-associated glycoprotein complex were the most significantly enriched cellular components. The most common molecular functions among the DEGs were heparan sulfate proteoglycan binding, intracellular chloride channel activity, intracellular calcium-activated chloride channel activity, proteoglycan binding, EMC structural constituent, transforming growth factor beta binding, nitric-oxide synthase binding, fibronectin binding, ankyrin binding, and calcium-release channel activity.

The results of the KEGG enrichment analysis demonstrated that ECM-receptor interaction, focal adhesion, vascular smooth muscle contraction, proteoglycans in cancer, the cGMP-PKG signaling pathway, the PI3K-Akt signaling pathway, regulation of the actin cytoskeleton, human papillomavirus infection, pathways in cancer, and several other associated KEGG biological pathways were significant to the progression of PCa (Figure 3D).

### Extraction of Metabolism-Related Genes From the GSEA Website

We acquired 41 metabolic pathway gene sets from MSigDB and KEGG (Supplementary Table 2). To identify the metabolism-related genes in PCa, we examined the enrichment scores of 41 metabolic pathway gene sets by using GSEA, extracting 948 metabolism-related genes (Supplementary Table 3).

### Identification of the Candidate Genes and Confirmation of the Five Optimal Genes

To identify common genes among those we extracted above, all three gene groups, including direct PPP1R12A protein interactors, PPP1R12A-related DEGs, and metabolism-related genes, were compared. Although we failed to identify a candidate gene among all three parts of independent gene sets, we selected overlapping candidate genes from the intersection of every two independent gene sets, respectively. In total, 15 overlapping elements in total were identified between the PPP1R12A-related DEGs and the metabolism-related genes (Supplementary Figure 1A). In the comparison of the PPP1R12A-related DEGs and the interactive genes, two overlapping genes were identified. Seven overlapping elements were found in the comparison between the metabolism-related genes and the interactive genes (Supplementary Table 4). To construct the prognostic signature efficiently, we identified four optimal genes (*PTGS2*, *GGCT*, *AOX1*, and *NT5E*) from the candidate genes we extracted previously through Scikit-learn’s random forest classifier (Supplementary Figure 1B). These five genes (including *PPP1R12A*) were used to build a predictive signature.

### Construction and Verification of the Prognostic Model Based on the Five-Gene Signature

**FIGURE 4:**
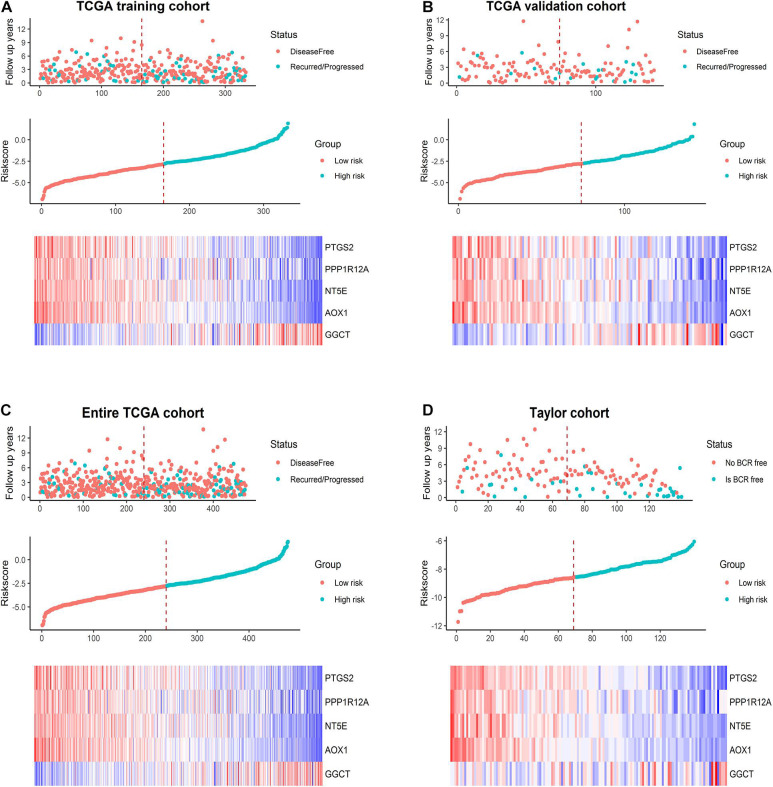
Prognostic model analysis based on the five-gene signature. Analysis of the survival status distribution, risk score, and heatmap based on the five-gene signature in **(A)** the TCGA training set, **(B)** the TCGA validation set, **(C)** the entire TCGA cohort, and **(D)** the Taylor cohort.

We calculated the RS for each PCa patient in the TCGA training set and ranked them to thoroughly analyze the relevance of the five promising genes in prognosis in these patients. The regression coefficients of the five optimal genes in the Cox regression analysis are shown in Supplementary Table 5. Thus, those patients were classified into a low-risk group (*n* = 167) and a high-risk group (*n* = 166) based on median RS (cut-off = –2.7737) (Figure 4A, middle). The survival status in the training set is shown in Figure 4A, top. Additionally, the five promising genes were differentially expressed in the high- and low-risk groups, as shown in the heatmap in Figure 4A, bottom. Utilizing data from the TCGA validation set, the entire TCGA set, and the Taylor database, the RS was calculated in each cohort with the same formula as in the TCGA training set. We confirmed the prognostic value of the five-gene signature by confirming our findings from the TCGA training set (Figures 4B–D).

**FIGURE 5:**
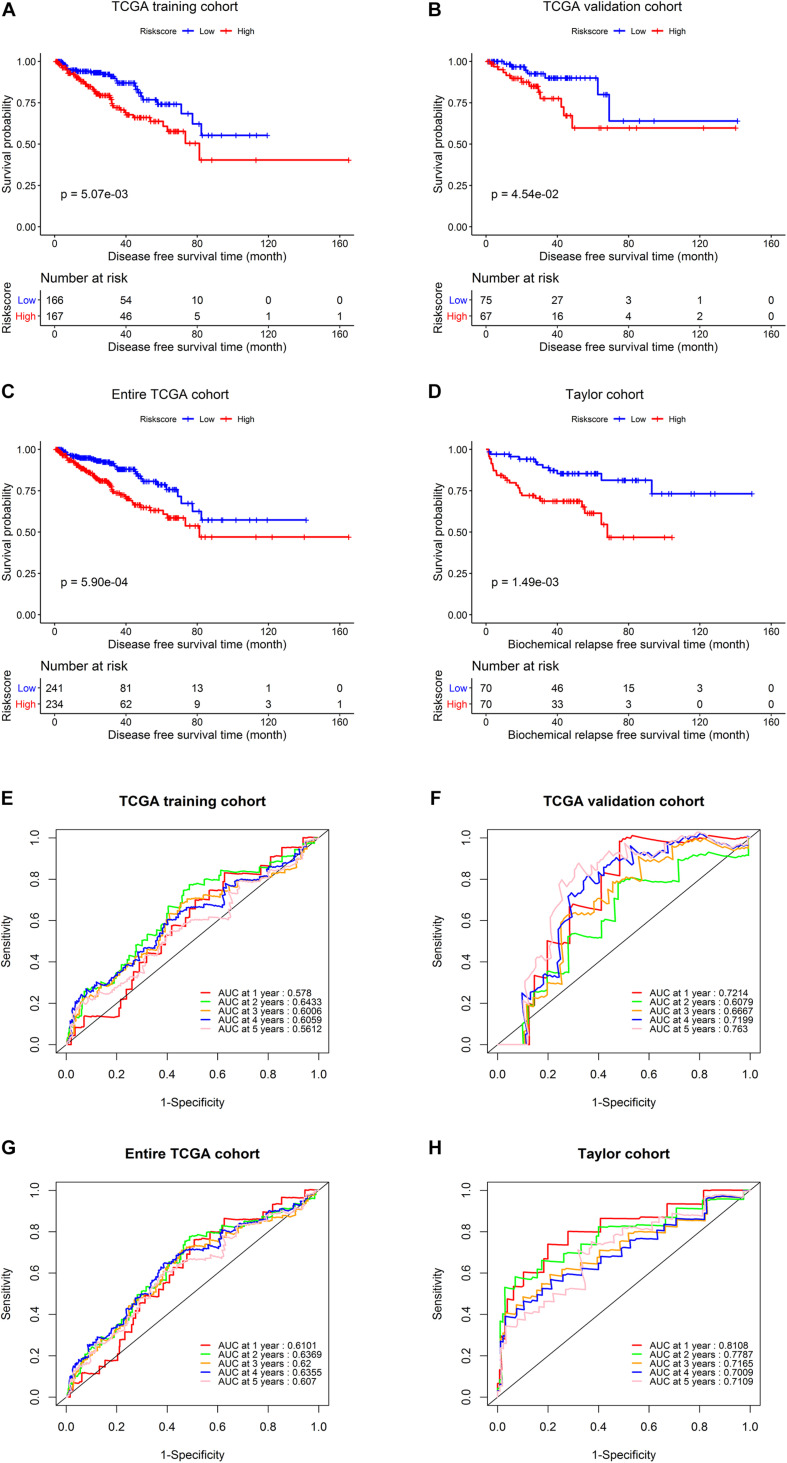
Survival analysis and predictive value validation of the five-gene signature. Comparison of DFS stratified by risk group in the TCGA dataset and comparison of BRFS between the low- and high-risk groups in the Taylor dataset. ROC curves testing the predictive value of the risk score in the following four cohorts: **(A,E)** TCGA training set, **(B,F)** TCGA validation set, **(C,G)** entire TCGA cohort, **(D,H)** Taylor cohort.

Next, we conducted Kaplan–Meier analysis. In the TCGA training set, patients in the high-risk group exhibited significantly worse DFS than those in the low-risk group (Figure 5A) (*p* = 0.00507). Similar analyses of the Kaplan–Meier curve (Figure 5B) showed that compared to the high-risk group, patients in the low-risk group exhibited a significantly better DFS in the internal validation set (*p* = 0.0454). In the entire TCGA set of 475 patients, patients in the high-risk group suffered worse DFS than patients in the low-risk group (*p* = 0.00059, Figure 5C). Consistently, patients in the low-risk group generally had a better BRFS than patients in the high-risk group in the Taylor external validation cohort (*p* = 0.00149, Figure 5D). Time-dependent ROC curve analysis revealed that the five-gene signature had a strong predictive ability in internal and external datasets (Figures 5E–H).

### Expression Profiles of the Five-Gene Signature and Subgroup Analysis

**FIGURE 6:**
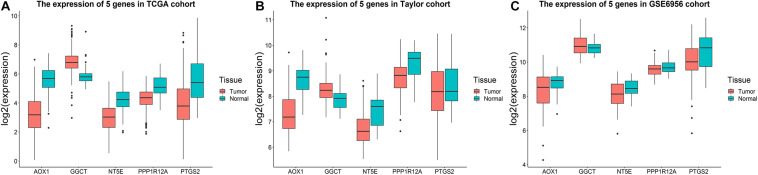
Expression profiles of the five-gene signature. Boxplots presenting the expression levels of the five promising genes (*AOX1*, *GGCT*, *NT5E*, *PPP1R12A*, and *PTGS2*) in the tumor and tumor-free groups from **(A)** the TCGA cohort, **(B)** the Taylor cohort, and **(C)** GSE6956.

Utilizing the data concerning different tissues in three databases, we explored the five genes’ expression profiles. As shown in Figure 6A, *GGCT* expression was significantly upregulated in tumor tissues, while *AOX1*, *NT5E*, *PPP1R12A*, and *PTGS2* were significantly downregulated compared to normal tissues in the TCGA cohort. Similar results were obtained in the Taylor and GSE6956 datasets (Figures 6B,C). Subgroup analysis showed that the five-gene signature-based RS had a good predictive ability for DFS in different subgroups, including tumor-free patients (*p* = 0.00825), patients with tumor (*p* = 0.0322), patients aged < 65 years (*p* = 0.0238), patients aged ≥ 65 years (*p* = 0.00866), patients with a Gleason score of < 8 (*p* = 0.0173), patients with a Gleason score of ≥ 8 (*p* = 0.0488), patients with stage T1/T2 PCa (*p* = 0.000615), patients with stage N0 PCa (*p* = 0.000604), and patients with stage M0 PCa (*p* = 0.00107), in the TCGA cohort (Supplementary Figure 2).

## Discussion

PCa is one of the most common malignant urinary tumors, threatening human health globally. Few effective therapeutic strategies are available for patients with advanced or metastatic diseases, especially CRPC. Consequently, potential biomarkers of PCa that can be used to improve prognostic assessment are urgently needed. We previously identified that PPP1R12A could inhibit angiogenesis and tumor growth, and has been identified as a promising prognostic factor for human PCa. Moreover, previous studies (Dong et al., 2017) showed that cancer metabolism is an essential process in tumorigenesis, while research about the relation between metabolism and the tumor microenvironment in PCa is limited. Therefore, it is important to explore new biomarkers that interact with PPP1R12A for early effective prognosis and personalized treatment of PCa. Thus, we performed a TAP-MS analysis to identify direct PPP1R12A interactors on a proteomic scale, using full-length PPP1R12A protein as bait. We identified 51 direct human PPP1R12A interactors.

First, we conducted an integrated analysis of TCGA datasets to investigate the underlying biomarkers interacting with PPP1R12A in metabolism in PCa. Then, a total of 340 PPP1R12A-related DEGs, including 338 upregulated and 2 downregulated genes, were obtained between 52 normal tissues and 499 PCa tissues. Next, GO analysis and KEGG pathway enrichment analysis were conducted. Calcium-activated chloride channel and chloride channel activity are related to the regulation of cell proliferation, cell migration, and metastasis and are proposed to contribute to tumor growth and invasion in several cancers, including PCa (Lang and Stournaras, 2014; Hu et al., 2019). In addition, EMC (Walker et al., 2018) and cell adhesion (Odero-Marah et al., 2018) are connected to progression and metastasis of PCa. Beyond that, KEGG pathway analysis revealed that these DEGs were primarily involved in vital signaling pathways, including the MAPK signaling pathway, the PI3K-AKT signaling pathway, ECM-receptor interaction, focal adhesion, the cGMP-PKG signaling pathway, and actin cytoskeleton regulation. It was reported that PI3K-AKT signaling is upregulated in PCa, and CRPC is associated with excessive activation of the PI3K-AKT pathway (Carver et al., 2011; Crumbaker et al., 2017). As for the ECM, a vital structural element of the tumor microenvironment, dysregulation of the ECM–receptor interaction signaling pathway was related to the regulation of tumor invasion and metastasis (Zhang et al., 2016; Yeh et al., 2018). Moreover, another study implied that focal adhesion was related to tumor occurrence and metastasis (Eke and Cordes, 2015). Wang et al. (2020) found that the cGMP-PKG signaling pathway also has a tight relationship with proliferation, migration, and invasion in PCa.

After overlapping and Scikit-learn’s random forest classifier analysis, *PTGS2*, *GGCT*, *AOX1*, *NT5E*, and *PPP1R12A* were selected as optimal genes. Prostaglandin-endoperoxide synthase 2 (PTGS2), also known as cyclooxygenase-2(COX-2), a crucial enzyme in the process of arachidonic acid conversion to prostaglandins and other eicosanoids, was reported to promote malignant metastasis in colorectal tumor cells (Fenwick et al., 2003). In addition, Guo et al. (2020) demonstrated that PKM2 upregulates COX-2 expression, leading to epithelial-mesenchymal transition (EMT) and metastasis of PCa, by promoting the phosphorylation of ERK1/2. More studies on the role of COX-2 in the regulation of PCa metastasis are required. According to a previous study (Kageyama et al., 2018), γ-glutamylcyclotransferase (GGCT), an indispensable enzyme in connection with glutathione metabolism, is upregulated in most cancers, including PCa. GGCT deficiency leads to the suppression of proliferation, invasion, and migration of cancer cells. It has been demonstrated that downregulation of aldehyde oxidase 1 (AOX1) in PCa is related to shorter times until biochemical recurrence (Li et al., 2018). Of note, 5′-nucleotidase ncto (NT5E), also called CD73, whose expression levels can be used to effectively distinguish between aggressive types and indolent forms of PCa, was demonstrated to be a potential independent prognostic marker (Leclerc et al., 2016). The present study provides a direction for further research on the biological functions and clinical characteristics of the five genes in PCa.

Finally, a five-gene prognostic model was established using the TCGA training cohort, and patients were divided into high-risk and low-risk groups. The AUC values for predicting the 1–, 2–, 3–, 4–, and 5-year DFS rates were 0.578, 0.643, 0.601, 0.606, and 0.561 in the TCGA training cohort, respectively, demonstrating that the model is a reliable predictor of prognosis. We used the data from the TCGA validation cohort and the external Taylor cohort to confirm the prognostic superiority of the five-gene signature. Survival analysis in the validation set confirmed our results from the training set, demonstrating that our five-gene risk model was robust. Additionally, the prognostic significance of the signature was analyzed in different subgroups in the TCGA cohort. The signature also showed a good predictive ability for DFS in different PCa patient subgroups (based on TNM stage, Gleason score, age, or patient status), verifying that the five-gene signature is a functional marker independent of other clinicopathological features.

Despite the merits of the present study, certain limitations should be acknowledged to avoid its overinterpretation. First, it is difficult to fully evaluate the quality of the data used in the study because the number of samples acquired from the databases was relatively low, and part of the gene expression data and clinical data involved were retrieved from open databases. Secondly, further in-depth experimental studies should be conducted to investigate the biological functions and clinical characteristics of the five genes identified in this study.

## Conclusion

In conclusion, based on publicly available data and our experimental results, we established a robust five-gene signature to predict the prognosis of PCa by stratifying PCa patients into high-risk and low-risk groups. Its prognostic value was validated in an internal cohort from the TCGA database and an external cohort from GEO. Subgroup analysis confirmed that the signature is a functional marker independent of other clinical features. We hope that the signature will guide treatment strategies for PCa patients.

## Data Availability Statement

The datasets presented in this study can be found in online repositories. The names of the repository/repositories and accession number(s) can be found in the article/Supplementary Material.

## Ethics Statement

The studies involving human participants were reviewed and approved by the Ethics Committee of Guangzhou First People’s Hospital. The patients/participants provided their written informed consent to participate in this study.

## Author Contributions

YuL and WZ conceived, designed the study and revised the manuscript. ZZ, YF, and YiL analyzed the data. RL, QD, YaZ, and ZH contributed to programming. MJ, XZ, SC, and JL collected the clinical samples and data. RZ, ZT, and YiZ performed the experiments. ZZ and YuL wrote and edited the draft. All authors contributed to the article and approved the submitted version.

## Conflict of Interest

The authors declare that the research was conducted in the absence of any commercial or financial relationships that could be construed as a potential conflict of interest.

## Publisher’s Note

All claims expressed in this article are solely those of the authors and do not necessarily represent those of their affiliated organizations, or those of the publisher, the editors and the reviewers. Any product that may be evaluated in this article, or claim that may be made by its manufacturer, is not guaranteed or endorsed by the publisher.

## Footnotes

1
http://ualcan.path.uab.edu/

2
https://portal.gdc.cancer.gov/

3
https://xenabrowser.net/

4
http://www.cbioportal.org/

5
https://www.ncbi.nlm.nih.gov/geo/

6
https://www.gsea-msigdb.org/gsea/index.jsp

7
https://scikit-learn.org/stable/
