# Supplementary material for: Identification and Validation of a PPP1R12A-Related Five-Gene Signature Associated With Metabolism to Predict the Prognosis of Patients With Prostate Cancer
Source: Front Genet. 2021 Aug 13;12:703210. doi: 10.3389/fgene.2021.703210 (PMC8414655; doi:10.3389/fgene.2021.703210)
Supplement: Supplementary file 1 [file Data_Sheet_1.ZIP › Supplementary Material/Supplementary_Figures.docx]

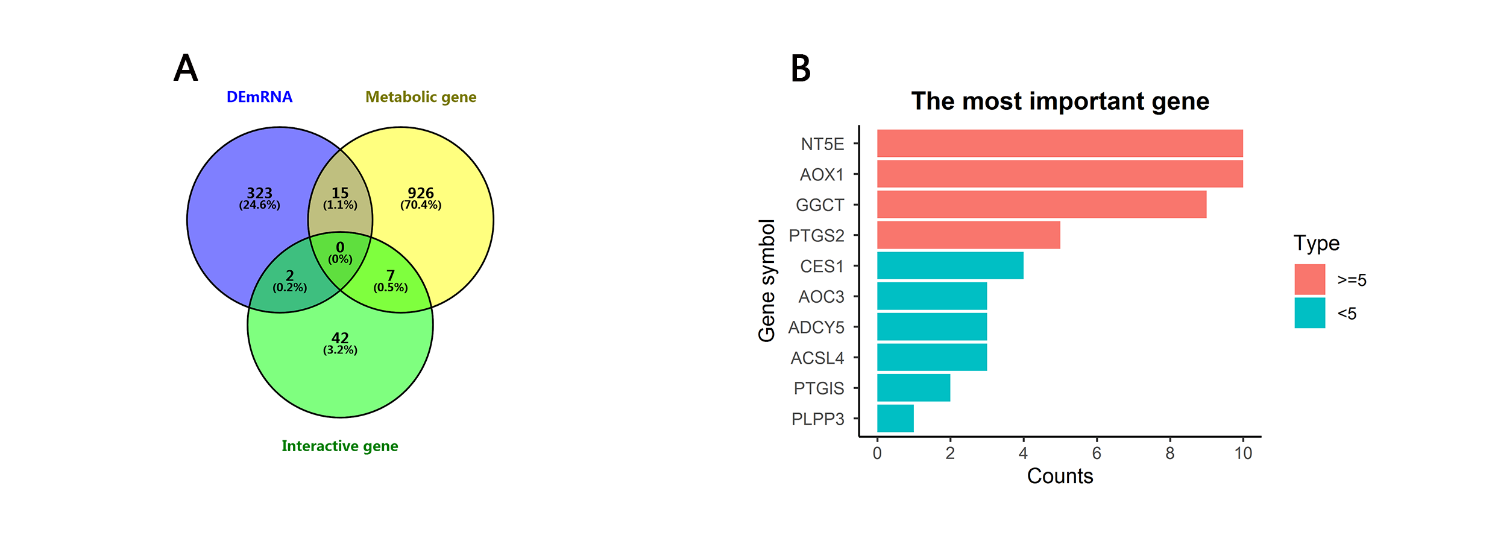


**Supplementary** **Figure 1.** Identification of the candidate genes.

**(A)** Venn diagram of overlapping candidate genes from the intersection of every two independent gene sets, respectively. Overlapping areas represent common genes between different gene sets. **(B)** Histogram of the counts of being top 5 important gene rank by Scikit RF. Red bar mean the genes which appeared not less than five times in the top five important genes rank.


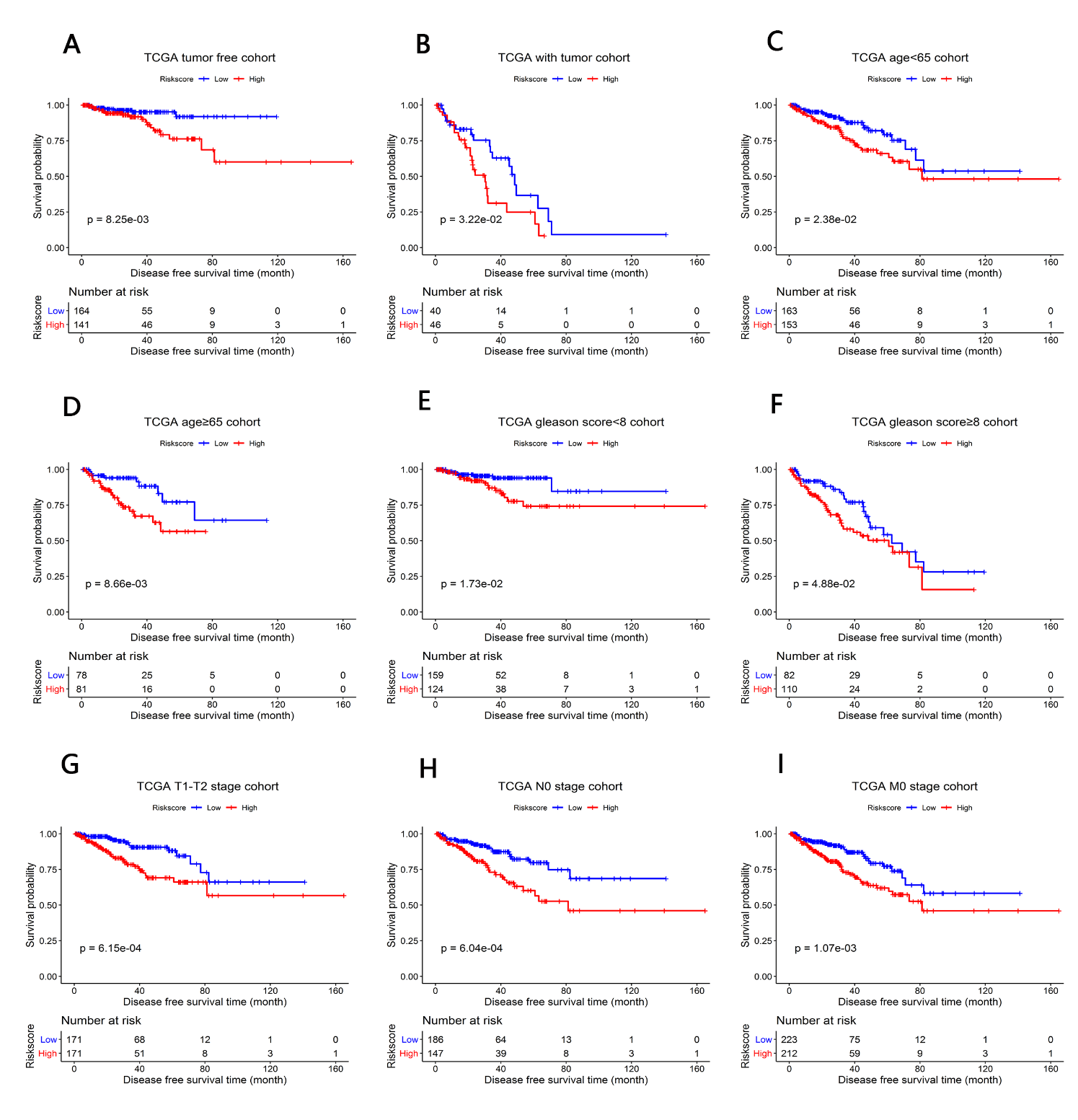


**Supplementary** **Figure 2.** Survival analysis in subgroups of PCa patients based on the five‑gene signature.

Signature-based risk score is a reliable marker for DFS in subgroups, including clinical status **(A, B)**, age **(C, D)**, gleason score **(E, F)**, tumor stage **(G-I)**.
